# A Novel Device to Exploit the Smartphone Camera for Fundus Photography

**DOI:** 10.1155/2015/823139

**Published:** 2015-06-02

**Authors:** Andrea Russo, Francesco Morescalchi, Ciro Costagliola, Luisa Delcassi, Francesco Semeraro

**Affiliations:** ^1^Eye Clinic, Department of Neurological and Vision Sciences, University of Brescia, Piazzale Spedale Civili 1, 25123 Brescia, Italy; ^2^Eye Clinic, Department of Health Sciences, University of Molise, Via de Santis, 86100 Campobasso, Italy

## Abstract

*Purpose*. To construct an inexpensive, convenient, and portable attachment for smartphones for the acquisition of still and live retinal images. *Methods*. A small optical device based on the principle of direct ophthalmoscopy was designed to be magnetically attached to a smartphone. Representative images of normal and pathological fundi were taken with the device. *Results*. A field-of-view up to ~20° was captured at a clinical resolution for each fundus image. The cross-polarization technique adopted in the optical design dramatically diminished corneal Purkinje reflections, making it possible to screen patients even through undilated pupils. Light emission proved to be well within safety limits. *Conclusions*. This optical attachment is a promising, inexpensive, and valuable alternative to the direct ophthalmoscope, potentially eliminating problems of poor exam skills and inexperienced observer bias. Its portability, together with the wireless connectivity of smartphones, presents a promising platform for screening and telemedicine in nonhospital settings. *Translational Relevance*. Smartphones have the potential to acquire retinal imaging for a portable ophthalmoscopy.

## 1. Introduction

Retinal imaging has considerably improved since the first photographic images of the ocular fundus were taken near the end of the 19th century [1].

Traditionally, this approach has relied upon expensive and bulky tabletop units, operated by a trained technician in a hospital/clinic setting. These units are complex optical assemblies that require patients to be seated upright, which is often difficult for hospitalized or bedridden patients [2]. Portable fundus cameras have recently become commercially widespread, but these are often costly or remain difficult to use in an ergonomic, hand-held manner [3–5].

To overcome these limitations, we took advantage of physicians' pervasive adoption of smartphones, which are equipped nowadays with state-of-the-art cameras. We developed a small optical device, which is attached magnetically to a smartphone, to conveniently examine and record videos or photographs of the retina. This attachment, which we call D-Eye, leverages the portability and wireless connectivity of current smartphones, making it possible to acquire retinal pictures even in remote areas. In this paper, we present this portable and inexpensive solution, while demonstrating its feasibility in a variety of clinical settings, for the acquisition of retinal images at clinical resolution.

## 2. Materials and Methods

**Figure 1:**
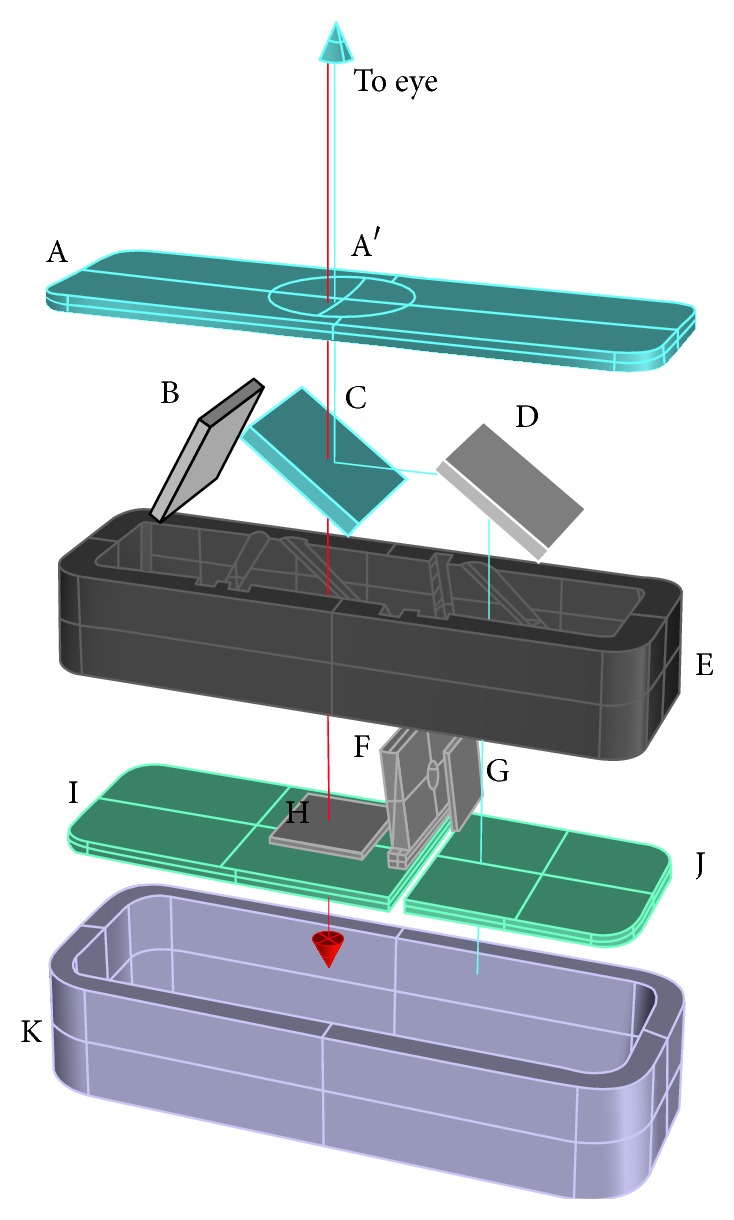
Exploded view of the D-Eye module (angles and distances between components are approximated). Retinal images are acquired using coaxial illumination and imaging paths thanks to a beam splitter (C). The blue arrow depicts the path of the light; red arrow depicts the path of fundus imaging. Device components are glass platelet (A) with imprinted negative lens (A′), photo-absorbing wall (B), beam splitter (C), mirror (D), plastic case (E), diaphragm (F), polarized filters (G, H), flash and camera glass (J, I), and magnetic external ring (K).

D-Eye works on the principles of direct ophthalmoscopy and exploits the smartphone camera's autofocus capability to account for a patient's refractive error. A front negative lens (Figure 1, A′) is imprinted in a glass plate, which serves as the top cover nearest the eye. This lens shifts the focus of the smartphone from infinity to −8 cm, in order to exploit the smartphone's autofocus range of about 18 diopters. This allows for a compensation of refractive error from −12 to +6 diopters. All plastic components and slots were designed using the Rhino 3D software (MCNeel, Seattle, WA), exported to a stereolithography file format, and then 3D-printed using a Replicator 2 3D printer (Makerbot, New York, NY). The final prototype measures approximately 47 × 18 × 10 mm and weighs 7 grams. The mechanics and the optical path are shown in Figure 1. Briefly, the light emitted by the “flash” light-emitting diode (LED) is conveyed into the eye by a mirror (Figure 1, D) and a beam splitter (Figure 1, C), as with a direct ophthalmoscope. The diaphragm (Figure 1, F), the polarizing filters (Figure 1, G and H), and the photo-absorbing wall (B) remove reflections and flares; otherwise they present in such a configuration. This cross-polarization design also eliminates corneal Purkinje reflections.

**Figure 2:**
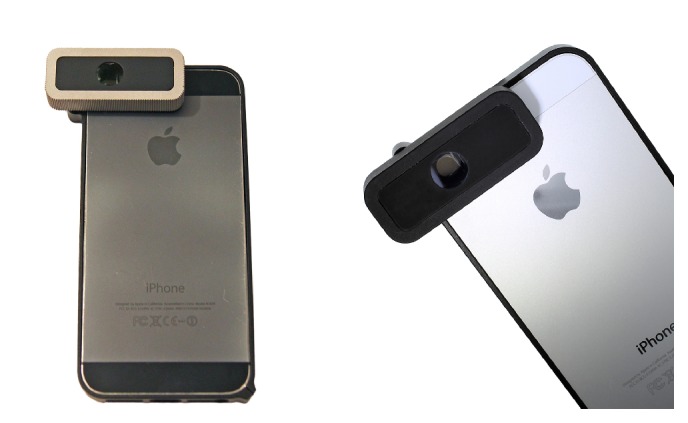
Picture of the prototypes magnetically attached to different iPhone (5 and 5s; Apple, Cupertino, CA) models.

The smartphone requires no modification, and a bumper fitted with properly located neodymium magnets (N45) ensures the correct alignment of the device with the smartphone's camera and flash (Figure 2).

### 2.1. Retinal Imaging

**Figure 3:**
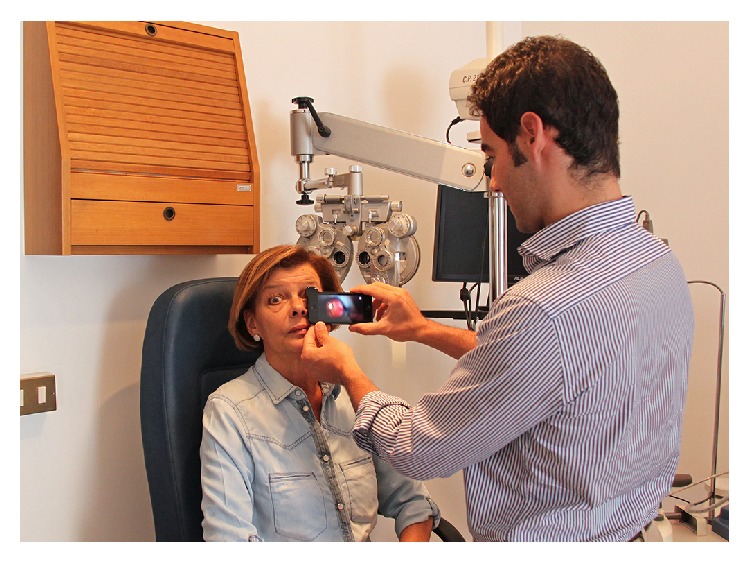
The acquisition procedure is similar to traditional direct ophthalmoscopy, but the examiner can work from a comfortable position.

We developed a smartphone application that enables dimming the intensity of the flash LED and switching between auto- and manual focus (the latter being required for undilated pupils). Acquisition is conducted similarly to traditional direct ophthalmoscopy by placing the system at a distance of ~1 cm from the patient's eye. However, the examiner can work from a comfortable position—with no need to lean toward the patient's face—by aiming through the smartphone's screen (*a video of the acquisition procedure is attached; Figure 3*). Acquired images or videos can subsequently be saved in the local memory or stored via secure server. D-Eye is designed to work with Galaxy S4 and Galaxy S5 (Samsung, Seoul, South Korea) and iPhone 5, iPhone 5s, and iPhone 6 (Apple; Cupertino, CA). In this study, we coupled the module with an iPhone 5.

### 2.2. Clinical Testing Protocol

Clinical testing of the device was performed at the Eye Clinic of the University of Brescia after approval by the Institutional Review Board and in full compliance with the Declaration of Helsinki. All participants in the study provided written, informed consent. Fundus photography was carried out both before and after pharmacologic pupil dilation by using 0.5% tropicamide and 10% phenylephrine.

## 3. Results

**Figure 4:**
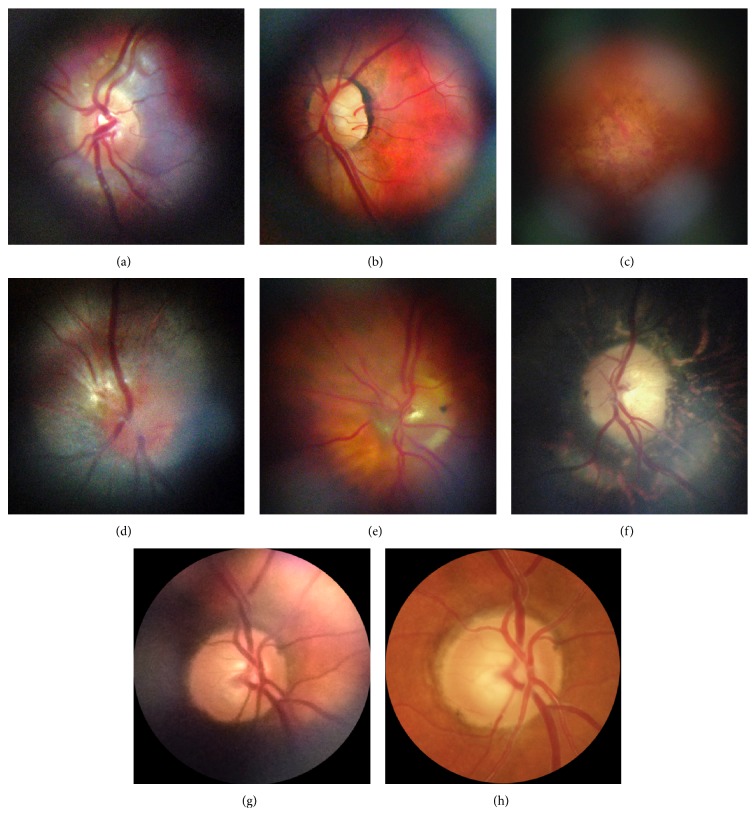
Representative retinal images taken with D-Eye. (a) Normal optic disc in an undilated child. (b) Normal posterior pole in a dilated 29-year-old woman. (c) Dry age-related maculopathy in an undilated 75-year-old man. (d) Optic nerve glioma in a 23-year-old undilated woman. (e) Posterior vitreous detachment in a dilated 72-year-old pseudophakic woman. (f) Waxy disc pallor and pigmentary changes in a 50-year-old man with retinitis pigmentosa. ((g) and (h)) Depiction of the same optic nerve head by D-Eye and Canon CR-2 Retinal Camera.

Smartphone-based ophthalmoscopy, performed with the module described earlier, captures high-quality retinal images. Representative retinal images taken with the D-Eye system are shown in Figure 4.

When operating through a dilated pupil, the system captures a field-of-view up to ~20 degrees for a single fundus image at a distance of 1 cm from the patient's eye; the exact angular field varies as a function of pupil diameter. This field aperture is much wider than that of direct ophthalmoscopes (usually 5–8 degrees) and is comparable with that of the iExaminer (Welch Allyn, Skaneateles Falls, New York).

Images are captured by the 3264 × 2448-pixel camera sensor, using ~150 pixels per retinal degree. This considerably exceeds the image resolution benchmarks of 6 M pixels and 30 pixels per degree, set forth by the United Kingdom's National Health Service for effective retinopathy screening and detection of DR-related pathology [5].

Smartphone ophthalmoscopy with the D-Eye proved to be ergonomic, being performed in a hand-held manner, regardless of whether the patient was standing, sitting, or lying down. The smartphone can be held with one hand while the other guides the patient's fixation. From the beginning of the procedure, the time required to capture a video or burst of still images is less than 1 minute. Subject variability in media opacities and pupil diameter were found to significantly affect the overall quality of the pictures, making acquisition difficult for pupil diameters <2.5 mm.

**Figure 5:**
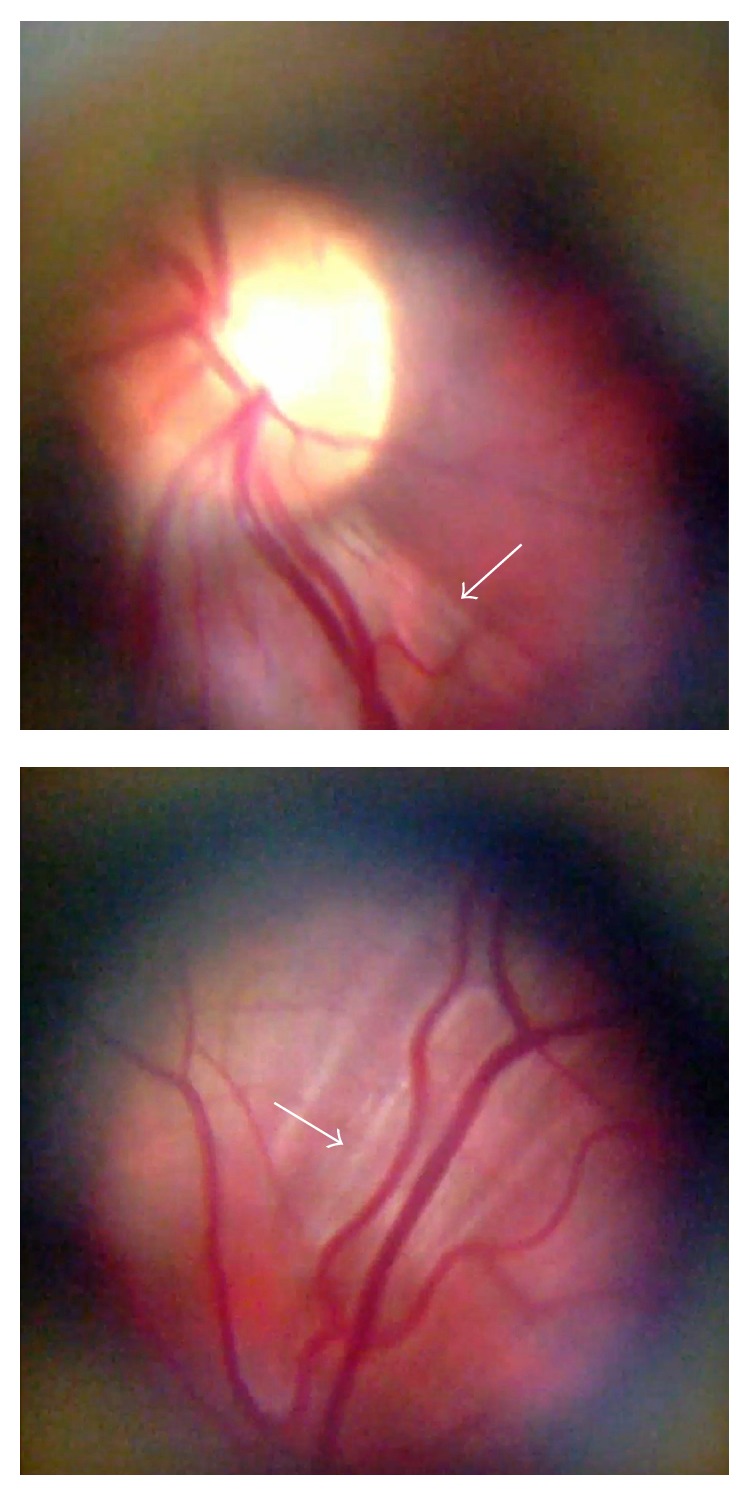
Cross-polarization-accentuated nerve-fiber layer definition (white arrows).

The cross-polarization technique adopted in the optical design resulted in a dramatic minimization of artifacts and reflections and allowed for a complete reduction of corneal Purkinje reflection, allowing patients to be screened through undilated pupils (Figures 4(a) and 4(d)). In addition, the cross-polarization technique improved image detail and contrast and increased the definition of the nerve-fiber layer by reducing its reflectivity (Figure 5) [6].

### 3.1. Safety

The light safety limits for ophthalmic instruments are set by the International Organization for Standardization (ISO 15004-2.2). These safety limits are at least one order of magnitude below actual retinal threshold damage. The irradiance of the iPhone 5 LED's light, dimmed with the D-Eye application and conveyed through the diaphragm, polarizing filter, and diverging lens, was 3.2 mW/cm^2^, which is 220 times below the thermal limit (706 mW/cm^2^). For photochemical hazard, the weighted retinal radiant exposure was 32 mJ/cm^2^ (exposure duration of 1 minute), which is 312 times below the photochemical limit (10 J/cm^2^). The high electronic sensitivity of the smartphone's camera compensates for the low emission of light into the eye, which is more than 10 times less than that of a commercial Keeler indirect ophthalmoscope [7].

## 4. Discussion

Recent literature emphasizes smartphones as valuable tools in the field of ophthalmology, while they are also beginning to play a central role as medical diagnostic tools in general [4, 8, 9]. In fact, owing to the portability, data storage capability, and wireless connectivity of smartphones, it is plausible that a smartphone's fundus camera could soon play a significant role in clinical settings. Furthermore, it is estimated that more than one out of every two physicians already uses a smartphone [10].

The D-Eye module is compact, extremely portable, and capable of performing retinal imaging at clinical resolution and, thanks to its design, can fit a number of smartphones by replacing its magnetic bumper.

An inherent ergonomic ease makes this smartphone ophthalmoscopy technique easier than traditional direct ophthalmoscopy, since the examiner does not need to lean in toward the patient but can work at a convenient distance, using the smartphone's screen to focus its camera on the patient's eye. Moreover, owing to the relatively low hardware and production costs, the D-Eye's retail price would be less than $400, making the device suitable for community vision screening by a variety of nonophthalmic medical personnel.

A recent comparative instrument study assessed the accuracy and reliability of smartphone ophthalmoscopy and showed a considerable agreement with dilated retinal biomicroscopy for the grading of diabetic retinopathy (simple *κ* = 0.78; CI 0.71–0.84) [11].

Thanks to the absence of corneal reflections, only a few seconds are needed to visualize the optic disc with undilated pupil, creating the conditions for a worthwhile screening, particularly for glaucoma. Moreover, we noticed an amazing convenience in the assessment of babies, since they seem to be spontaneously attracted by the nondisturbing light emitted by the device, making the fundus acquisition straightforward.

The beta version of the D-Eye application we developed can record a burst of still images or a video of the fundus. A stitching algorithm to pan across the entire posterior pole and the peripheral retina is currently under development.

The advantages of smartphone-based retinal image acquisition in remote, nonhospital settings include portability and immediate upload/analysis. Indeed, telemedicine has the potential to reach patients and communities that currently receive negligible or suboptimal eye care as a result of geographic or sociocultural barriers, or both [12].

In conclusion, this attachment for smartphones might be a promising alternative to the direct ophthalmoscope, as its portability and wireless connectivity present strong potential applications such as telemedicine, even in nonhospital or rural settings.
